# The association between community environment and cognitive function: a systematic review

**DOI:** 10.1007/s00127-014-0945-6

**Published:** 2014-08-03

**Authors:** Yu-Tzu Wu, A. Matthew Prina, Carol Brayne

**Affiliations:** 1 Department of Public Health and Primary Care, Institute of Public Health, Forvie Site, School of Clinical Medicine, University of Cambridge, Cambridge Biomedical Campus, Cambridge, CB2 0SR UK; 2 Health Service and Population Research Department, Centre for Global Mental Health, Institute of Psychiatry, King’s College London, De Crespigny Park, Denmark Hill, London, SE5 8AF UK

**Keywords:** Cognitive function and dementia, Systematic review, Community environment, Neighborhood

## Abstract

**Purposes:**

The aim of this study is to review the published evidence on the association between community environment and cognitive function in older people, focusing on the findings and a critique of the existing studies.

**Methods:**

A literature search was conducted to identify studies linking the community environment and cognitive function in older people. The results and methodological factors, including the definition of community, individual level characteristics and the measurements of cognitive function and community environment were extracted from each study. The measurements of community environment were mainly categorized into two types: compositional, generated by aggregating individual and household data (community-level socioeconomic status, deprivation index) and contextual, targeting at the features of built or social environment in local areas (green space, street conditions, crime rate).

**Results:**

Fourteen of the fifteen studies used compositional measurements such as community-level socioeconomic status and deprivation index and significant associations were found in eleven studies. Some individual level factors (ethnicity, genotype and socioeconomic status) were found to modify the association between community environment and cognitive function. Few contextual measurements were included in the existing studies. A conceptual framework for the pathway from community environment to cognitive function of older people is provided in this review.

**Conclusions:**

To disentangle the additional effect of place from individual risk factors and investigate the casual direction of community environment and cognition in later life, longitudinal studies with measurements targeting built and social environments of community and change of cognitive functions over time need to be included in future studies.

## Introduction

The quality of living environment in communities has been considered as a substantial determinant of health [1]. The community can be regarded as a psychosocial factor in an intermediate position between individual health and broad influences of social contexts [2]. Recent decades have seen an increased interest in studying the association between community environment and health of local residents. Examining the environmental features of communities might throw light on potential risk factors in the living environment and pathways by which social factors might determine health.

### The health of older people and community

The older population is considered to be a vulnerable group that is likely to be affected by a poor community environment, given that this group spends a significant amount of time in the community and it is more dependent on local resources and services [3]. As a number of studies have reported that community environments might have a major role in supporting ageing populations, providing friendly living environments for older people is a substantial public health issue, which should be on the agenda of both local authorities and national governments [4, 5]. An evidence-based approach is needed to understand the interactions between community and health conditions among older people.

Several studies have examined the influence of community environment on the health of older people, focusing on general health, physical activity, obesity and depression [3, 6–9]. The literature of environmental gerontology also suggests that there is a potential impact of neighborhood deterioration, poor quality of physical and social environment on the health and well-being of older people [10–12]. However, the measurements of community environment considerably varied between different studies. Many studies have used compositional measurements, which are generated by aggregating individual characteristics or residents’ perceptions of their environments in defined geographical units, as robust measurements of the community (Table 1). Community-level socioeconomic status, which combines several individual or household socioeconomic variables, was widely used in earlier studies to measure neighborhood deprivation and the association with health outcomes. This type of measurement is strongly correlated with individual characteristics and causes difficulties in disentangling the additional effect of living environment on health from the influence of individual risk factors. Perceived measurements can lead to “same-source bias”, meaning that people who are healthier, more physically and socially active are more likely to report positive attitudes to community environment than those who are less active [13].

**Table 1:** The types of measurements used to assess the characteristics of community

Type	Measure	Content	Data source
Compositional	Community-level Socioeconomic status	Townsend deprivation index (households without car, overcrowded, not owner-occupied, unemployment) % of household poverty % of unemployment % of homeownership % of adults over 25 without high school degree % of adults with professional or managerial occupation	National statistics and census
	Perceived social environment	Crime and safety Community network and cohesion Social disorders (drugs problems, public drunkenness)	Questionnaire, interview, national survey
	Perceived built environment	Satisfaction to living environments and local services	Questionnaire and interview
Contextual	Social environment	Collective efficacy (voter turnout, crime rate) Social organization Ethnicity fragmentation (Index of Dissimilarity)	National survey and statistics, Yellow page
	Built environment	Safety (features of disorders and urban designs) Public open space/greenness Walkability (street connectivity, land use mixed) Food environment and local resource (recreation centers, food stores, library, church, café)	National statistics, GIS, Yellow page, direct observation and investigation,

On the other hand, contextual measurements, which target the features of built environment (the actual setting and environment of the community) and social environment (community networks, organizations and reputations) in communities, have become more prominent in recent studies. Several new instruments have been developed to capture physical features of places, such as walkability, greenness and natural environment, availability of public open areas and parks, food environments, local services and land use [14]. Many studies have examined their effects on physical activity and mobility in older people [6, 7]. However, only few studies have been able to avoid using perceived measurements to investigate the influence of community environment on the mental health illnesses of older people [9].

### Cognitive function: an important mental health issue in later life

As cognitive frailty and dementia are important aspects of mental health and quality of life in older people, it is important to consider potential determinants beyond individual level risk factors and synthesize existing evidence. Several risk factors for dementia, such as stress, negative emotions, lack of physical activity and social networks have also been associated with poor designs of community and unsafe living environments [15–18].

Geographical variations in dementia prevalence indicate that the characteristics in local areas might have potential influence on cognitive frailty in older age [19]. The association between community environment and cognitive impairment has been investigated in some studies but no systematic review which synthesizes the strengths and limitation of this literature exists as yet.

### Aims of the study

The aim of this study is to review the studies that examined the association between community environment and cognitive function in later life, focusing on the findings and limitations of the existing studies.

## Methods

### Search strategy and selection criteria

A literature search was conducted in PubMed, Embase and Web of knowledge to identify the studies related to cognitive function (cognitive impairment, cognitive decline, and dementia) and community environment (neighbourhood/neighborhood, living environment, and residential environment) until February 2014. Title screening was first conducted to exclude irrelevant studies and the abstracts of potential articles were reviewed by two separate reviewers (YTW, AMP) based on three inclusion criteria:

1. Cognitive function was the main health outcome
2. Community was defined as a geographical area close to the participants’ place of residence
3. The studies measured the characteristics of community

**Fig. 1:**
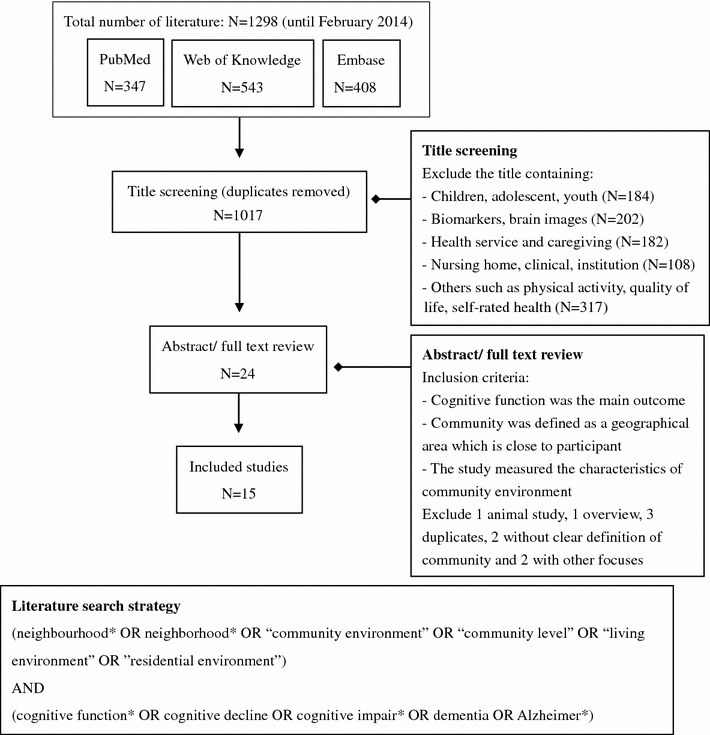
Flow diagram of literature search

The studies that examined cognitive frailty in regional or area level (cities, provinces and countries), or focused on cognitive function in children and adolescents were excluded from this review. More detailed information on the literature search is provided in Fig. 1.

### Data collection

The results and methodological factors, including the definition of community, the measurement of cognitive function and community environment, the characteristics of participants and individual level confounders were extracted from each study. The measurements of community environment were mainly categorized into two types: compositional, generated by aggregating individual characteristics in defined community units, and contextual, which targets the features of places rather than people. Individual level confounders were categorized into four types: Demographics (D), such as age, sex, marital status, ethnicity; Individual socioeconomic status (SES), including education, occupation, social class, income; Health status (HS), including several chronic diseases such as hypertension, diabetes, stroke, depression; Health behaviors (HB), including smoking, alcohol drinking, physical activity.

## Results

Fifteen studies, which examined the association between community environment and cognitive function in older population, were identified and included in this review [20–34]. No studies examined the association between community environment and dementia. Ten studies were conducted in the US, two in the UK, one in The Netherlands, one in China and one in Singapore.

Compositional measurements were included in 14 studies but none of them included perceived measurements [20–24, 26–34]. Few contextual measurements were included in the studies. Four studies discussed the interaction of individual conditions and living environment. Table 2 summarized the methods and measurements of the 15 studies.

**Table 2:** Studies which examined the association between community and cognitive function

Reference	Setting	Method	Participants	Age	Measures	Community definition	Community measurements	Individual controls^a^	Mediator/modifier	Statistical analysis	Results^d^
Boardman et al. [23]	US, Chicago, Chicago health and ageing project	Cross-sectional (1993)	1,665 people living in Chicago	65+	MMSE, memory, perceptual speed	Boundaries created by the local authority	Compositional: Neighborhood disadvantage, social disorders	D, education	Modifier: disadvantage genotype	Multilevel analysis	Inter (APOE genotype)
Clarke et al. [24]	US, city of Chicago, Chicago community adult health study	Cross-sectional (2002)	949 adults living in Chicago city	50+	TICS	2000 US census tracts	Compositional: Neighborhood disadvantage, neighborhood affluence Contextual: Neighborhood resources: recreational centers, institutions; neighborhood disorder	D, SES, HS	Mediators: social integration, civic engagement, physical activities;	Multilevel analysis	C-SES (−, *β* = 0.28) Contextual (resource: +, *β* = 1.81)
Sisco and Marsiske [31]	US, 6 areas, Advanced cognitive training for independent and vital elderly	Cross-sectional (1997–2000)	2,802 people	65–94	Multiple cognitive functions^b^	2000 US census tracts	Compositional: Neighborhood socioeconomic position	D, quadratic age, education		Multilevel analysis	C-SES (−, *β* = 0.07)
Wee et al. [32]	Singapore	Cross-sectional (2012)	909 people in two residential sites	60+	Cognitive impairment (MMSE)	Blocks	Compositional: Area socioeconomic status	D, SES, HS		Multilevel analysis	C-SES (+, OR = 3.8)
Al Hazzouri et al. [20]	US, California; Sacramento Area Latino study on aging	Longitudinal, 7 waves (1998–2008)	1,789 Mexican American	60–101	Cognitive decline (3MSE)	2000 US census tracts	Compositional: Neighborhood socioeconomic context	D, SES, HS		Multilevel analysis (3 levels)	C-SES (−, *β* = 0.01)
Aneshensel et al. [21]	Heath and retirement survey	Cross-sectional at time 3 (1996)	4,525 nationally representative	70+	TICS	1990 US census tracts	Compositional: Socioeconomic disadvantage, racial segregation	D, SES, HS, social integration	Modifiers: ethnicity (African American and Hispanic)	Multilevel analysis	C-SES (−, *β* = 0.04) Inter (ethnicity)
Lee et al. [28]	US, Baltimore The Baltimore memory study	Cross-sectional (2001)	1,124 people from 65 neighborhoods of Baltimore	50–70	Multiple cognitive functions^b^	2000 US census tracts	Compositional: Social disorganization, economic deprivation Contextual: Public Safety, physical disorder	D, SES, HB, numbers of siblings and children, retirement, job control	Modifier: disadvantage genotype	Multilevel logistic analysis (2 levels)	Inter (APOE genotype)
Shih et al. [30]	US, Women’s health initiative memory study	Cross-sectional at baseline (1996)	1,342 women	65–81	3MSE	Combined 1990/2000 US census tracts	Compositional: Neighborhood socioeconomic status	D, SES, HB, HS		Multilevel analysis	C-SES (−, *β* = 0.02)
Wen and Gu [33]	China, 22 provinces, Chinese longitudinal healthy longevity survey	Cross-sectional^d^ (baseline 2002) Longitudinal (health outcomes 2005)	8,099 people	65–79	Cognitive impairment (MMSE)	County or city district	Compositional: Average years of schooling, labor force participation rate, proportion of urban population Contextual: Per capital GDP, Number of hospital beds per 1,000 persons	D, HB, SES, pollution index, Childhood SES^c^		Multilevel analysis	C-SES (+; OR: 1.4)
Sheffield and Peek et al. [29]	US, 5 southwestern states, Hispanic established population for epidemiologic studies of the elderly	Longitudinal, 3 waves (1993–1999)	1,980 Hispanic people	65+	Cognitive decline (MMSE)	1990 US census tracts	Compositional: Economic advantage, social disadvantage	D, SES, HS		Multilevel analysis (2 and 3 levels)	C-SES (+, OR = 1.8)
Basta et al. [22]	UK, 5 centers MCR cognitive function and ageing study	Cross-sectional (1992)	13,004 people	65+	Cognitive impairment (MMSE),	Census 1991 Postcodes mapped to enumeration district	Compositional: Townsend deprivation score	Sex, SES, centers		Multilevel analysis	C-SES (+, OR = 2.3)
Lang et al. [27]	UK, England, The English longitudinal study of ageing	Cross-sectional (2002)	8,102 urban-based adults	50+	MMSE	Census 2001; super output area	Compositional and contextual: Index of Multiple Deprivation 2004	D, SES, HB, HS		Ordinary least square regression	C-SES (−, *β* = 0.18)
Wight et al. [34]	US, Study of assets and health dynamic among the oldest old	Cross-sectional (1993)	3,442 people in urban setting	70+	TICS	1990 US census tracts	Compositional: Neighborhood education and median household income	D, SES, HS		Multilevel analysis	C-SES (−, *β* = 1.76)
Deeg and Thomese [25]	The Netherlands, Longitudinal aging study Amsterdam	Cross-sectional (1992)	2,981 people	55–85	MMSE	Postcode	Contextual: Neighborhood income status: rental price of rented houses, purchase price of owner-occupied houses, the monthly household income of a sample family	D, SES, living periods	Modifier: individual income status	Linear regression	Inter (individual income)
Espino et al. [26]	US, San Antonio longitudinal study of aging	Cross-sectional (1992)	452 Mexican and 375 European Americans	65+	Cognitive impairment (MMSE)	Neighborhood (Barrio, transitional, suburban)	Compositional: Barrio (low-income, Mexican–American) Transitional (middle-income, mixed ethnicity) Suburbs (high-income, adapted culture)	D, SES, HS	Modifier: ethnicity (Mexican and European Americans)	Logistic regression	Inter (ethnicity,)

Results: *C-SES* Community-level socioeconomic status (more vs less deprived), *Inter* interaction with individual factors, *+* positive association, *−* negative association, *OR* odds ratio, *β* effect size of regression coefficient

^a^Individual controls: *D* demographics, including age, sex, marital status, ethnicity, nativity; *SES* individual socioeconomic status, including education, occupation, social class, income; *HS* health status, including hypertension, diabetes, stroke, depression, arthritis, heart attack, fall in the past year, hearing impairment; *HB* health behaviors, including smoking, alcohol drinking, physical activity

^b^ *Multiple cognitive functions* language, processing speed, eye-hand coordination, executive function, verbal memory and learning, visual memory, visuoconstruction (Lee et al. [28]); memory, reasoning, processing speed, everyday cognition, vocabulary (Sisco and Marsiske [31])

^c^ *Childhood SES* measured by urban-born, father white-collar job, parents alive at age 10, access to health care, hungry, arm length

^d^More detailed results of cross-sectional associations at baseline 2002 were reported in Zeng et al. [47]

Ten studies used the Mini-mental State Examination (MMSE) or modified Mini-mental State Examination (3MSE) to measure cognitive function, three used the Telephone Interview for Cognitive Status (TICS) and two included multiple tests of cognitive function. Two studies had a cut-off score to define “cognitive impairment” and two considered the decline of cognitive functions over time [20, 22, 29, 33]. Most studies defined communities using census-related units, such as census tracts in the US and output areas in the UK.

### Community-level socioeconomic status and deprivation

The majority of studies used community-level socioeconomic status and deprivation indexes (compositional measurements) as robust measures of socioeconomic disadvantage and deprivation in the community. These measurements were similar across the studies and were usually considered as a combination of individual or household socioeconomic variables in census data, such as the percentage of poverty, homeownership, adults without high school degree and families with a single parent.

Eleven studies focused on the association between community-level socioeconomic status, area deprivation and cognitive function of older people. Nine studies measured the characteristics of community by aggregating individual data in the census. Eight of them presented significant associations between local deprivation and poor cognitive function [21, 30, 31, 34], cognitive decline [20, 29] or higher risk of cognitive impairment [22, 32]. Living in more deprived areas was associated with higher odds of cognitive impairment or decline (range 1.4–3.8). Due to the heterogeneity of the measures across the studies, it is difficult to summarize the effect sizes of the associations between area deprivation and cognitive function. Detailed effect sizes in individual studies are reported in Table 2.

Two studies used other measurements to describe broader socioeconomic status in local areas. The English Longitudinal Study of Ageing used the Index of Multiple Deprivation 2004 (IMD 2004), which not only combined several domains of compositional measures (income, employment, education, training, health and disability) but also added contextual deprivation (barrier to housing and service, living environment and crime) [27]. The Chinese Longitudinal Healthy Longevity Survey defined the socioeconomic status of community by five statistics based on the administrative boundaries. This included GDP per capital, labor participation rate, proportion of urban population, numbers of hospital beds per 1,000 people and average schooling years [33]. Both studies found significant associations between high area deprivation, poor cognitive function and cognitive impairment.

### Interaction of individual state and living environment

Some individual characteristics have been found to modify the association between living environment and cognitive function. High-risk genotype of dementia (apolipoportein E, APOE), individual income status and ethnicity were three factors which have been examined in the literature.

Two studies investigated gene-environment interactions, including socioeconomic disadvantages and perceived (social disorders) or objective measurements (public safety, physical features) of disorder [23, 28]. An independent association of social disorder on cognitive function was found, with an interaction between psychosocial hazard, social disorders and high-risk genotype, which associated with lower cognitive function, also reported. One study explored the potential issue of “relative deprivation” using matched or discrepant income status between neighborhoods, defined by rental and purchase price of houses, and individual socioeconomic status [25]. Discrepant neighborhood and personal income status was found to have an association with worse cognitive ability. Two studies examined the modified effect of ethnicity on the association between community-level socioeconomic status and cognitive function in later life [21, 26]. In both studies, the disadvantaged environment was significantly associated with poor cognitive ability in ethnic minority groups.

### Contextual measurements

Few studies included contextual measurements, taking into account the features of built and social environment in the community [24, 25, 33, 34]. The independent effect of contextual measurements was rarely explored in existing studies. Most of these studies combined contextual with compositional measurements, as a synthesized measure of the characteristics of community environment. One smaller study included some contextual resources (recreational centers, libraries, churches, schools) and neighborhood disorders (present of graffiti, litter, broken glasses) from the census area. It is also the only study that investigated potential mechanisms, reporting that the association between neighborhood socioeconomic structure (the composition of residents) and individual cognitive function could be mediated by contextual resources, recreational facilities and local services [24].

## Discussion

### Main findings

Fifteen studies have reported on the association between community environment and cognitive function of older people. Fourteen of them used compositional measurements such as community-level socioeconomic status and deprivation index. Significant associations were found in eleven studies with various measures of community and cognitive function. Seven studies reported a negative relationship of area deprivation and cognitive function. Positive associations between community-level socioeconomic disadvantage, cognitive impairment and cognitive decline were reported in four studies with an odds ratio ranging from 1.4 to 3.8. Some individual risk factors including high-risk genotype of cognitive frailty, individual income status and ethnicity were found to effect modifiers on the association between living environment and cognitive function. Very few contextual measurements were included in the existing studies.

### Limitations of the existing studies

The limitations of using community-level socioeconomic status or deprivation index as a key measurement of community are manifest in the existing studies. Although multilevel modelling can take the variation within and between the communities into account, these compositional measurements were actually generated from individual data. The strong correlations between individual and community level measurements mean it is almost impossible to adjust for individual level risk factors completely and separate the effect of “place” from “people”. To identify the additional effect of place from residual influence of individual factors properly, it is necessary to fundamentally improve study design and both carefully consider the measurements of community environments and the incorporation of advanced statistical methods.

Some of the environmental measurements might not reflect the real community living conditions. For example, local GDP per capita is influenced by the industry and business activity in the area but might not be directly related to the life of local people. The quality of built environment (such as maintenance of pavements and public properties) and social environment (such as local social networks, neighborhood watch) could be more related to living conditions in communities. Without clear hypotheses and rationales to specify potential influences of environmental features on cognitive function, the contextual measurements used in the existing studies were generally considered parts of community-level socioeconomic status or deprivation index, rather than independent variables representing the characteristics of community environment. Although many studies found a significant association between community-level socioeconomic status and cognitive function, the effect of community environment on cognitive function is still ambiguous. The pathway from “poor community” to “poor cognition” or vice versa is difficult to explore and the key features within community environments that might influence cognitive function in older people are still unknown. Some studies indicated an interaction between community environments and individual characteristics. However, most of them used fixed-effects regression models to analyze their data. These methods might not appropriately deal with the variations within and between communities and overestimate strength of the association [35].

Most studies were of a cross-sectional nature, which might indicate potential reverse causality between a poor living environment and cognitive functions. People with cognitive impairment may be more likely to live in deprived environments because of their poor health status and lack of economic ability. Although decline in cognitive function is more likely to happen in later stages of life and people usually start settling down in one area during mid-life, the specified effect of community environment on cognitive function cannot be answered comprehensively by cross-sectional studies alone [25, 36].

The association between community environment and dementia has not been reported in the existing studies. People with dementia are more likely to move to institutions or care homes and have higher mortality. It is more difficult to approach this population and collect environmental data before they moved to institutions or died. Small sample sizes might lead to non-significant results and potential publication bias.

### From community environment to individual cognitive function

**Fig. 2:**
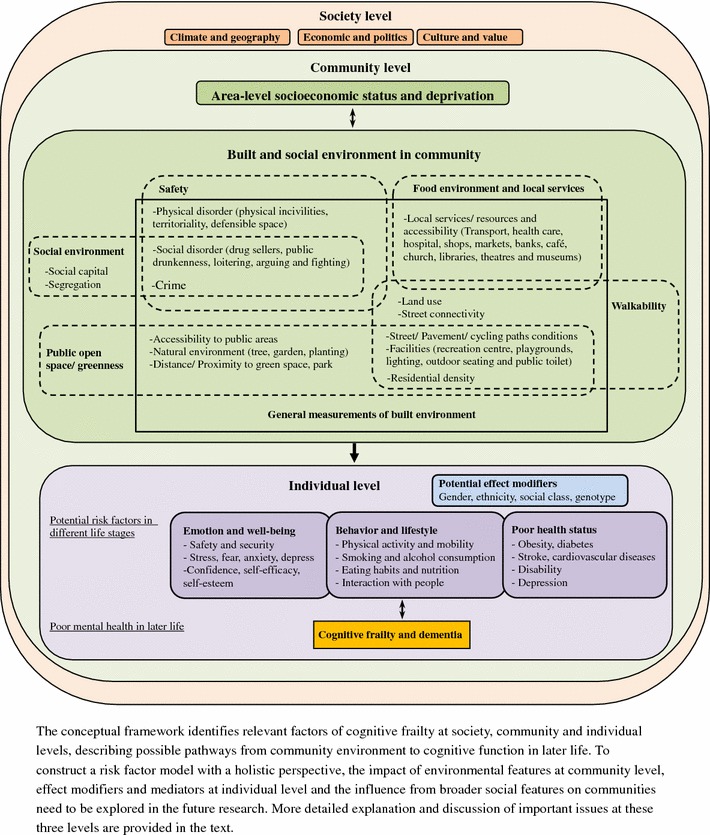
Conceptual framework of the pathway from community environment to cognitive function of older people

For non-communicable diseases and mental health, it is important to construct risk factor and determinants models with a holistic perspective. A recent review has proposed causal pathways from community environment to depression and indicated the importance of both direct and indirect influences on individual mental health [37]. Unfavorable conditions in community environments could not only cause stress and perception of lack of control but also increase the risk of depression through deteriorating supportive social networks. Similarly, the complicated relationship of community environment and cognition in later life needs to be explored and considered in detail. Figure 2 is a conceptual framework which identifies relevant factors of cognitive frailty at society, community and individual levels, describing possible pathways from community environment to cognitive function in later life. The following discussion focused on important issues at these three levels:

#### Community level: from “local deprivation” to “environmental features”

There is some evidence supporting that the variation of cognitive impairment at a community level can be explained by local deprivation. Considering possible additional effects of place on health, this could indicate the indirect pathway from “poor people”, “poor place” to “poor health outcomes” or that the compositional measurements might only be a proxy for the quality of built and social environments in community [13, 24]. Both pathways highlight the importance of investigating specific environmental features at community level to provide more information on better living environments for healthy aging. Based on findings reported in both social and health science, important built and social environmental features related to physical and mental health at older age are summarized in Fig. 2 and categorized into different groups [7, 8, 14]. These measurements might also have substantial impacts on cognitive function in later life through different pathways.

Built environmental features, such as green space, access to local services and basic infrastructures (lighting, street and path conditions) are considered to be fundamental elements of supporting active and healthy ageing [4, 5]. Older people could be deprived of basic activities, social interactions and cognitive stimulation due to poor quality of built environment in communities. Neighborhood deterioration, social disorder and crime have been linked to lack of control in living environments, stress and poor mental health with potential influence on emotional and cognitive health in later life through psychosocial pathways [8, 37]. From a life-course perspective, risk factors in younger and mid-life stages might have various influences on cognitive function in older age [38]. Cumulative stress and long-term distress due to poor quality of community environment could influence cognitive function in later life.

#### Individual level: potential mediators and effect modifiers

Exploring possible mediators will be important in understanding the complicated mechanism of community-level features to individual cognitive functions in later life. As several studies found that the features of built and social environment in communities are related to emotion, well-being, behavior and lifestyle of residents in middle and later life, these individual level factors could be potential mediators to poor cognitive function in older age [3, 7, 9]. For example, lack of physical activity, which is a risk factor for several chronic diseases, cognitive impairment and dementia, is found to be associated with poor quality of the built environment and might be considered as a mediator of cognitive frailty.

Besides mediators, the effect modification of individual demographic factors will be important in moderation of the relationship between environmental features and individual cognitive frailty (Fig. 2). The same physical features in a living environment might have various influences on different ethnicity groups, gender and social class and cause different impacts on health [26, 39]. Although collective interactions between people and their living environments are substantial, it might not be appropriate to use perceived measurements due to the strong correlation with individual mental health status. The two studies which were excluded from this review due to unclear community definitions, measured individual perception to social environment in their subjectively defined neighborhoods [40, 41]. The findings suggest a direct relationship and potential impact of social environment in communities on cognitive function of older population. However, these studies did not successfully investigate the influence of community-level measurements on individual health. Serious same-source bias could be an important issue considering the profound relationship of perception to environment, negative emotion and decline of cognition [13].

#### Society level: the influence of society on community environment

Some broader features of society as a whole, such as economics, politics and culture, might also influence built and social environments in communities as potential determinants of health in later life. In the UK and the US, recent policies and campaigns for addressing neighborhood safety and isolation of older people with the regeneration of local infrastructure are expected to have substantial influence on community environments and cognitive function in later life [42, 43]. Since cognitive decline is a chronic process, the long-term interaction between individual and community environment needs further consideration.

### Future research direction

Variation between the studies limits our ability to estimate a quantitative effect size and clarify causal directions. However, these are leads for new research since significant associations between community-level socioeconomic status, deprivation and cognitive function could represent the potential impact of community environment on individual cognitive function. To disentangle the effect of place from people, more contextual measurements need to be included to examine the influence of specific environmental features at community level. Some secondary data on small area level can be obtained from local government or national surveys. Methodologies to measure built environment in local area have been developed and supported by newer technologies, such as Geographical Information System (GIS), digital maps and images (Google Map, Google Street View, Bing Maps) [44]. For example, a recent study in Sweden used the GIS technique to assess the quality of green space and its association with mental health [45]. These tools can be used to develop new assessment methods and observe community environments in a more efficient and novel way. Direct observations of community environments can avoid using fixed administrative boundaries and collect primary data of various and detailed environmental features with a more flexible perspective.

To improve the disadvantage of cross-sectional studies and clarify causal direction, longitudinal studies with multiple time point measures of cognitive functions are desirable. To examine the long-term influence of community environments and dynamic interactions between people and place, it is important to measure the change of cognitive function in fixed populations since early older age and collecting environmental data of communities over time. Furthermore, the influence of community contexts on the health and well-being of individuals is considered to be especially important during certain periods of life, particularly childhood and old age [46]. It is necessary to integrate a life-course approach in longitudinal studies to insight into the interaction between individuals and community throughout life span and assist in developing public health strategies for healthy aging in place.
